# Characterization of *Pseudoterranova ceticola* (Nematoda: Anisakidae) larvae from meso/bathypelagic fishes off Macaronesia (NW Africa waters)

**DOI:** 10.1038/s41598-022-22542-0

**Published:** 2022-10-21

**Authors:** Miguel Bao, Kaja M. Olsen, Arne Levsen, Paolo Cipriani, Lucilla Giulietti, Julia E. Storesund, Eva García-Seoane, Egil Karlsbakk

**Affiliations:** 1 Institute of Marine Research (IMR), Nordnes, PO Box 1870, N-5817 Bergen, Norway; 2 Department of public Health and Infectious Diseases, Section of Parasitology, Sapienza University of Rome, Rome, Italy; 3 Department of Biological Sciences, University of Bergen, Bergen, Norway

**Keywords:** Parasitology, Biodiversity, Ecosystem ecology, Molecular ecology

## Abstract

The genus *Pseudoterranova* includes parasite species of cetaceans and pinnipeds. The third stage larva (L3) of seal-infecting species occur in second intermediate or paratenic fish hosts mainly in neritic waters. This study firstly describes a *Pseudoterranova* L3 from meso/bathypelagic fishes off Macaronesia. L3s were morphologically and genetically studied by light microscopy and sequencing of the mtDNA *cox*2 and entire ITS rDNA genes. Bayesian inferences were performed with sequences from the larvae and selected sequences from GenBank. The nematode L3s were molecularly identified as *Pseudoterranova ceticola,* a parasite of kogiid whales. Such larvae were collected from *Bolinichthys indicus*, *Chauliodus danae*, *Eupharynx pelecanoides*, *Diaphus rafinesquii*, *D. mollis*, *Diretmus argenteus* and *Maulisia argipalla*. They mainly occurred in the viscera of these fishes. *Pseudoterranova ceticola* L3 were small (< 12 mm) and whitish, and a prominent characteristic is a circumoral ridge extending from the ventral boring tooth which differentiate them from *Pseudoterranova* spp. L3 maturing in pinnipeds and *Terranova *sensu lato larvae that mature in poikilotherms. The shape of the tail: conical, long, pointed, ventrally curved and lacking mucron also distinguish these larvae from those of the pinniped-infecting *Pseudoterranova* spp. Phylogenetic analyses based on mtDNA *cox*2 and ITS rDNA sequences suggest that *P. ceticola* is closely related to *Skrjabinisakis* spp., and not with *Pseudoterranova* spp. parasitizing pinnipeds. The related species *Skrjabinisakis paggiae*, *S. brevispiculata* and *S. physeteris* (until recently belonging to genus *Anisakis*), are as *P. ceticola* also parasites of physeteroid cetaceans. The morphology and morphological variation of the larvae of the cetacean parasite *P. ceticola* is thoroughly described for the first time. These L3 can readily be morphologically distinguished from those of the pinniped-infecting *Pseudoterranova* spp. The parasite likely completes its life cycle in the mesopelagic and bathypelagic realm, with meso/bathypelagic fish as 2nd intermediate or paratenic hosts and kogiids as final host. Thus, *Pseudoterranova* from cetaceans appear to be morphologically, genetically, and ecologically differentiated to those from pinnipeds, suggesting that they are not congeneric.

## Introduction

The taxonomy of ascaridoid nematodes remains confusing and unresolved. The issue is of particular importance since species from the genera *Anisakis*, *Pseudoterranova* and *Contracaecum* are recognized as causative agents of fish-borne zoonotic diseases of worldwide concern, i. e. anisakidoses^1,2^. Generally, these anisakids use crustaceans as first intermediate hosts, fish and squid as second intermediate or paratenic hosts, and marine mammals (i. e. cetaceans for *Anisakis* spp. and pinnipeds for *Pseudoterranova* spp. and *Contracaecum* spp.) as final hosts of their life cycle [reviewed by 3]. In addition, anisakid species belonging to the genus *Terranova* (which is now considered taxon inquirendum, see^4^ and further comments at the discussion section) englobed species parasites of elasmobranchs, teleosts, crocodilians, colubrid snakes and marine mammals^4^.

Identification of *Terranova*-like third-stage larvae (L3) present in fish intermediate or paratenic hosts is difficult. Larvae belonging to *Pseudoterranova*, *Pulchrascaris* and *Terranova *sensu lato are too morphologically similar to identify them even to genus^5^. The common morphological features are the presence of the excretory pore opening ventrally at the anterior end, presence of ventriculus without an appendix and having an intestinal caecum^5–8^. Molecular identification is therefore needed. Robust identification of anisakid parasites is crucial for understanding their distribution and epidemiology.

In the present study, a new *Terranova*-like larval type, collected from mesopelagic/bathypelagic fish species of Macaronesia, North West (NW) African waters, is morphologically described, and molecularly recognized as a potentially zoonotic member of the genus *Pseudoterranova*.

## Methods

### Fish collection

**Figure 1:**
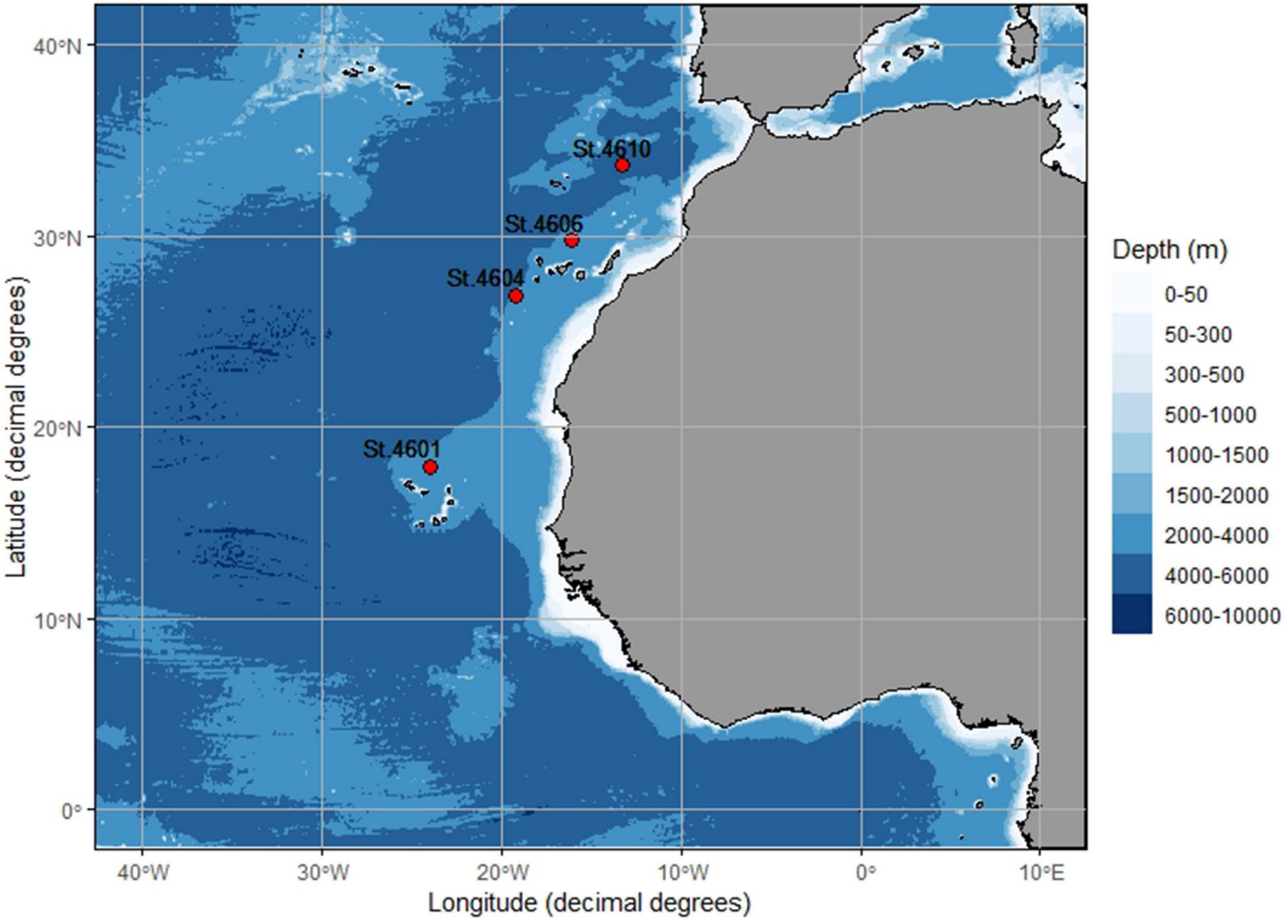
Trawl stations from which infected fish hosts were obtained. Positions: station (st.) 4601: 17.969 N, 23.956 W; st. 4604: 26.899 N, 19.232 W; st. 4606: 29.767 N, 16.087 W; st. 4610: 33.695 N, 13.232 W. Figure 1 was created using R version 4.95 (2021–03-31) (https://www.r-project.org/) implemented in RStudio 1.4.1106 (https://www.rstudio.com/) using.

During May 2019, mesopelagic and bathypelagic fishes including the following host species: *Bolinichthys indicus*, *Chauliodus danae*, *Eupharynx pelecanoides*, *Diaphus rafinesquii, Diaphus mollis, Diretmus argenteus* and *Maulisia argipalla* were caught in waters off NW Africa from Cape Verde to North East (NE) of Madeira during a research cruise on board of the Norwegian vessel “RV Kronprins Haakon” (Table 1, Fig. 1). Hauls were conducted with 2 different gears: a macroplankton trawl (theoretical mouth opening 6 × 6 and 8 mm stretched mesh size) and a Multipelt trawl (mouth opening height of 35 m and 20 mm mesh in the cod-end). Fishes were frozen on board at − 20 °C for later parasite inspection on land. Fish samples were collected within the MEESO project (EU H2020 research and innovation programme, Grant Agreement No 817669) and procedures were carried out in accordance with the relevant EU legislation including EU Directive 2010/63/EU of the European Parliament and of the Council of 22 September 2010 on the protection of animals used for scientific purposes. Norwegian research vessels have authorization to collect fishes for research purposes; in addition, permission for the collection of the present fishes was obtained from coastal countries.

**Table 1:** Overview of trawl stations from which the examined fish hosts were obtained, all in May 2019. *N* = number of *Terranova*-like larvae recovered per fish species and trawl station.

Station	Fish species	Day; starting time	Fishing gear	Max depth (m)
4601	*Diaphus rafinesquii* (*N* = 1)	03; 14:52	Macroplankton trawl	1650
4604	*Diaphus mollis* (*N* = 1)	07; 09:49	Multipelt trawl	1200
	*D. rafinesquii* (*N* = 23)			
	*Diretmus argenteus* (*N* = 2)			
	*Maulisia argipalla* (*N* = 1)			
4606	*Chauliodus danae* (*N* = 1)	09; 08:29	Multipelt trawl	1200
	*D. mollis* (*N* = 3)			
	*D. argenteus* (*N* = 1)			
4610	*Bolinichthys indicus* (*N* = 1)	13; 08:20	Macroplankton trawl	1200
	*Eupharynx pelecanoides* (*N* = 1)

### Parasite collection

Fishes were thawed at room temperature, opened and the visceral organs and emptied body cavity were placed in a petri dish with physiological saline solution and examined under stereomicroscope for ascaridoid nematodes. The parasites were collected, and the internal organs and carcass were then placed into plastic bags and refrozen. These were later examined using the UV-press method^9^, to detect any larvae not recovered during dissection, specially from the musculature.

### Morphological study

The nematode larvae were examined in temporary mounts in physiological saline solution, and photographed. Various morphotypes were recognized, but only findings concerning *Terranova*-like larvae^8,10^ (*N* = 35) will be presented here. In addition, infection levels such as parasite prevalence and abundance will be published elsewhere.

### Morphometric measurements

Series of digital photographs were obtained from 33 larvae. Measurements were taken from the digital images, except larval body lengths that mostly were measured at a mm scale.

**Figure 2:**
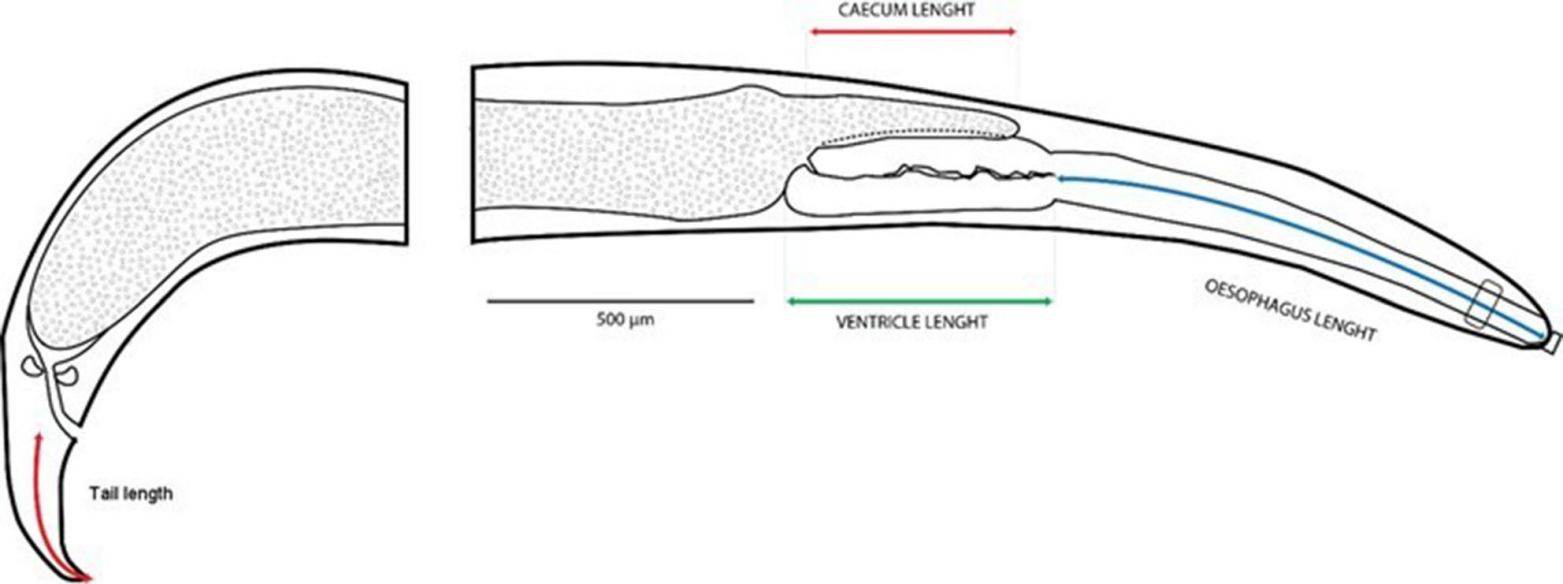
Measurements taken from the images of *Terranova*-like larvae. The oesophagus length is taken along the midline from the start of the oesophagus (i. e. slightly sub-terminally in the roundworm) to the ventricle. The caecum length was measured from the aperture into the ventricle to the caecum end. The tail length represents the distance along the midline, from the level of the anus/cloaca to the posterior end. Figure 2 was created in Adobe Photoshop 23.5.0 (https://www.adobe.com/photoshop).

Measurements from images were obtained using the software Image J (https://imagej.nih.gov/ij/). The oesophagus, ventricle and tail lengths were taken along the midline. The caecum length was measured from the aperture into the ventricle to the caecum end (Fig. 2).

### Molecular analyses

DNA was extracted from a randomly selected subsample of 19 nematode larvae using the DNeasy Blood & Tissue Kit (QIAGEN GmbH, Hilden, Germany) according to the manufacturer’s instructions with the modification that sample lysis was enhanced by mechanical disruption with ceramic bead-beating system (Precellys ceramic kit 2.8 MM, VWR and Precellys 24 Tissue Homogenizer, Bertin Technologies). DNA was eluted with 30 µl AE buffer.

Polymerase chain reaction (PCR) were done with primers that amplify the entire internal transcribed spacer (ITS) region of the nuclear ribosomal DNA (ITS1-5.8SrRNA -ITS2). The NC5F (5’–GTAGGTGAACCTGCGGAAGGATCATT- 3’) and NC2R (5’ TTAGTTTCTTTTCCTCCGCT -3’) primers were used. PCR conditions followed Zhu et al.^11^, but annealing temperature was 54 °C instead of 55 °C.

The mitochondrial cytochrome *c* oxidase subunit II (*cox*2) gene was amplified using the primers 211F (5′-TTTTCTAGTTATATAGATTGRTTTYAT-3′) and 210R (5′-CACCAACTCTTAAAATTATC-3′)^12^ according to Mattiucci et al.^13^ with the following modifications. The cycling conditions were an initial denaturation at 94 °C for 5 min, followed by 35 cycles of: denaturation at 94 °C for 30 s, annealing at 46 °C for 1 min, extension at 72 °C for 90 s; followed by final step of final extension at 72 °C for 10 min, and hold at 4 °C.

PCR products were sent for purification and sequencing (using the primers NC5F and 210R) to Eurofins (Cologne, Germany). The National Center for Biotechnology Information (NCBI) sequence database (henceforth ‘GenBank’) was searched for similar sequences using BLAST (Basic Local Alignment Search Tool) (USA)^14^.

### Phylogenetic analyses

Sequences generated in this study were aligned with selected sequences obtained from GenBank, using CLUSTAL W in MEGA X (Table 2 and 3)^15^. High similarity scores in the BLAST as well as larvae morphological similarity were used as the criteria to select the sequences. The default setting parameters of ClustalW were used, and the alignments were manually edited and trimmed in MEGA X. *Toxocara canis* and *Ascaris lumbricoides* were set as outgroup for the *cox*2 phylogenetic analysis. Due to indel-induced alignment problems in ITS^16^, only the closely related *Anisakis* spp., and *Pseudoterranova* spp. could be aligned with confidence in homology. For the same reason, no outgroup was included. The entire ITS sequences of *Pseudoterranova krabbei* and *Pseudoterranova bulbosa* identified from two cod (*Gadus morhua*) caught in northern Norwegian waters were sequenced and used in the analysis (Table 2).

**Table 2:** Samples used for analysis of the entire ITS rDNA.

Nematode species	GenBank accession number	Isolate	Stage	Host species	Location	References
Skrjabinisakis physeteris*	JQ912693	G	Adult	*Physeter macrocephalus*	Mediterranean Sea	Mattiucci et al.^13^
Skrjabinisakis brevispiculata*	JQ912694	H	Adult	*Kogia breviceps*	NW Atlantic Ocean	Mattiucci et al.^13^
Skrjabinisakis paggiae*	JQ912695	I	Adult	*K. breviceps*	NW Atlantic Ocean	Mattiucci et al.^13^
*Anisakis* sp. B	MK325217	21	Adult	*K. breviceps*	Australia	Shamsi et al.^65^
*Anisakis* sp.	JN005761	MAD17OI3	L3	*Pagellus bogaraveo*	Madeira	Hermida et al.^63^
*Anisakis* sp.	KC852170	10	Pre-adult	*Kogia sima*	Philippine	Quiazon et al.^57^
*Anisakis* sp.	None	Seq_A.sp.HC_2005	L3	*Ephinephelus areolatus*	Indonesia	Kleinertz et al.^81^
*Pseudoterranova decipiens (s. s.)*	AY825253	N241	Adult	*Phoca vitulina*	California	Nadler et al.^82^
*Pseudoterranova cattani*	KF781284	CL3	L3	*Homo sapiens*	Chile	Weitzel et al. ^83^
*Pseudoterranova azarasi*	AB576757	Pst-2	L3	*Gadus macrocephalus*	Japan	Arizono et al. ^84^
*Pseudoterranova decipiens sp. E*	KF017610	PDE2	L3	*Notothenia coriiceps*	Antarctica	Timi et al.^6^
*Pseudoterranova ceticola*	ON128286	SU6V1cet	L3	*Saurida undosquamis*	Tanzania	Cipriani et al. in prep
*Pseudoterranova krabbei*	OP355454	GMLOB31PF2	L3	*Gadus morhua*	Lofoten, Norway	This study
*Pseudoterranova bulbosa*	OP355455	GMHS76PV1	L3	*Gadus morhua*	Finnmark, Norway	This study
*Pseudoterranova ceticola*	OP352234	DiMo53T	L3	*Diaphus mollis*	Station 4606	This study
*Pseudoterranova ceticola*	OP352235	DiMo41T	L3	*Diaphus mollis*	Station 4606	This study
*Pseudoterranova ceticola*	OP352236	DiRa23T	L3	*Diaphus rafinesquii*	Station 4604	This study
*Pseudoterranova ceticola*	OP352237	DiArg15-13 T	L3	*Diretmus argenteus*	Station 4604	This study
*Pseudoterranova ceticola*	OP352238	DiRa37T	L3	*Diaphus rafinesquii*	Station 4604	This study
*Pseudoterranova ceticola*	OP352239	DiRa34-1 T	L3	*Diaphus rafinesquii*	Station 4604	This study
*Pseudoterranova ceticola*	OP352240	DiRa29 T	L3	*Diaphus rafinesquii*	Station 4604	This study
*Pseudoterranova ceticola*	OP352241	DiRa35-2 T	L3	*Diaphus rafinesquii*	Station 4604	This study
*Pseudoterranova ceticola*	OP352242	DiRa49T	L3	*Diaphus rafinesquii*	Station 4604	This study
*Pseudoterranova ceticola*	OP352243	DiRa38T	L3	*Diaphus rafinesquii*	Station 4604	This study
*Pseudoterranova ceticola*	OP352244	ChaDa53T	L3	*Chauliodus danae*	Station 4606	This study
*Pseudoterranova ceticola*	OP352245	EuPele 13 T	L3	*Eurypharynx pelecanoides*	Station 4610	This study
*Pseudoterranova ceticola*	OP352246	DiArg14-14 T	L3	*Diretmus argenteus*	Station 4604	This study

*Species formerly belonging to the genus *Anisakis* (see^20,21^).

**Table 3:** Samples used for analysis of the *cox*2 gene.

Nematode species	GenBank accession number	Isolate	Stage	Host species	Location	References
*Anisakis berlandi*	DQ116429		Adult	*Consensus sequence*		Valentini et al.^53^
*Anisakis pegreffii*	DQ116428		Adult	*Consensus sequence*		Valentini et al.^53^
*Anisakis simplex s. s*	KC810002	ASS1	Adult	*Balaenoptera acutorostrata*	Norway	Mattiucci et al.^13^
*Anisakis typica*	DQ116427		Adult	Consensus sequence		Valentini et al.^53^
*Anisakis ziphidarum*	DQ116430		Adult	Consensus sequence		Valentini et al.^53^
*Anisakis nascettii*	DQ116431		Adult	Consensus sequence		Valentini et al.^53^
Skrjabinisakis physeteris*	DQ116432		Adult	Consensus sequence		Valentini et al.^53^
Skrjabinisakis brevispiculata*	DQ116433		Adult	Consensus sequence		Valentini et al.^53^
Skrjabinisakis paggiae*	DQ116434		Adult	Consensus sequence		Valentini et al.^53^
*Anisakis typica*	KF701409	Ani1	Adult	*Tursiops aduncus*	Northern Red Sea	Kleinertz et al.^85^
*Skrjabinisakis* sp. 2*	MW074868	TMCRP20	L3	*Trachurus murphyi*	Peru	Aco Albuquerque et al.^86^
*Skrjabinisakis* cf. paggiae*	KF693770	AV60.8	Adult	*Kogia sima*	Brazil	Di Azevedo et al.^87^
*Anisakis sp. n. 1 KMAQ-2013 isolate 2*	KF214801	2	Adult	*Mesoplodon hotaula*	Philippine	Quiazon et al.^88^
*Pseudoterranova ceticola*	DQ116435			*Kogia breviceps*	Caribbean Sea	Valentini et al.^53^
*Pseudoterranova ceticola*	ON155434	SU6V1cet	L3	*Saurida undosquamis*	Tanzania	Cipriani et al.^19^
*Pseudoterranova ceticola*	LC712859	R2	L3	*Katsuwonus pelamis*	Japan:Mie, off Kumano	Takano & Sata^21^
*Pseudoterranova decipiens s. s*	MT347695	Pd03	L3	*Gadus morhua*	Lofoten (Norway)	Bao et al.^89^
*Pseudoterranova cattani*	KU558721			*Otaria byronia*	Chile	Liu et al.^90^
*Pseudoterranova bulbosa*	KU558720			*Erignathus barbatus*	Newfoundland	Liu et al.^90^
*Pseudoterranova azarasi*	MT912398	ZC17_335g		*Zalophus californianus*	California	Hrabar et al.^91^
*Pseudoterranova krabbei*	KU558724			*Halichoerus grypus*	Norway	Liu et al.^90^
*Pseudoterranova ceticola*	OP380493	Maar1T	L3	*Maulisia argipalla*	Station 4604	This study
*Pseudoterranova ceticola*	OP380494	DiMo53T	L3	*Diaphus mollis*	Station 4606	This study
*Pseudoterranova ceticola*	OP380495	DiMo41T	L3	*Diaphus mollis*	Station 4606	This study
*Pseudoterranova ceticola*	OP380496	DiRa23T	L3	*Diaphus rafinesquii*	Station 4604	This study
*Pseudoterranova ceticola*	OP380497	DiArg15-13 T	L3	*Diretmus argenteus*	Station 4604	This study
*Pseudoterranova ceticola*	OP380498	DiRa37T	L3	*Diaphus rafinesquii*	Station 4604	This study
*Pseudoterranova ceticola*	OP380499	DiRa34-1 T	L3	*Diaphus rafinesquii*	Station 4604	This study
*Pseudoterranova ceticola*	OP380500	DiRa29T	L3	*Diaphus rafinesquii*	Station 4604	This study
*Pseudoterranova ceticola*	OP380501	DiRa35-2 T	L3	*Diaphus rafinesquii*	Station 4604	This study
*Pseudoterranova ceticola*	OP380502	DiRa49T	L3	*Diaphus rafinesquii*	Station 4604	This study
*Pseudoterranova ceticola*	OP380503	DiRa34-2 T	L3	*Diaphus rafinesquii*	Station 4604	This study
*Pseudoterranova ceticola*	OP380504	DiRa36-1 T	L3	*Diaphus rafinesquii*	Station 4604	This study
*Pseudoterranova ceticola*	OP380505	DiRa38T	L3	*Diaphus rafinesquii*	Station 4604	This study
*Pseudoterranova ceticola*	OP380506	ChaDa53T	L3	*Chauliodus danae*	Station 4606	This study
*Pseudoterranova ceticola*	OP380509	DiRa22T	L3	*Diaphus mollis*	Station 4604	This study
*Pseudoterranova ceticola*	OP380508	DiArg7-1 T	L3	*Diretmus argenteus*	Station 4606	This study
*Pseudoterranova ceticola*	OP380510	DiRa37-3 T	L3	*Diaphus rafinesquii*	Station 4604	This study
*Pseudoterranova ceticola*	OP380507	DiArg14-14 T	L3	*Diretmus argenteus*	Station 4604	This study
*Ascaris lumbricoides*	AF179907			*Homo sapiens*	Louisiana	Nadler and Hudspeth ^12^
*Toxocara canis*	AF179923			*Canis familiaris*	Illinois	Nadler and Hudspeth ^12^

*Species formerly belonging to the genus *Anisakis* (see^20,21^).

Phylogenetic analyses were inferred using Bayesian inference (BI) method in BEAST v1.10.4^17^. The optimum evolutionary models were estimated using the Bayesian information criterion (BIC) as implemented in MEGA X. The optimum model was K2 + G for the ITS dataset based on BIC, independently of using all sites or complete deletion (i. e. gaps/missing data treatment). However, this model was not available in BEAST, so we used the best next best model available, i.e. HKY + G for the BI. The optimum model was HKY + G + I for *cox*2 dataset based on BIC criteria. The BEAST file was previously generated in BEAUti with the following characteristics: sites: entering the best substitution model and otherwise default settings; clock type: strict clock; tree prior: Speciation: Yule process; MCMC: length of chain = 10,000,000, echo state to screen every = 1000, log parameters every = 1000. Effective sample size of parameters (i. e. > 200) was checked in Tracer v.1.7.2^18^. The created tree was drawn in TreeAnnotator v1.10.4 and the burnin as the number of states was specified at 100,000. Figtree v1.4.4 was used to visualize the phylogenetic trees.

## Results

A total of 35 *Terranova*-type larvae were recovered. All these were morphologically similar. They were found in 7 fish species (Table 1). *In-situ*, the larvae were coiled like a coil or watch spring. Most (94%) were found in the viscera, but two larvae were found in the muscle of *D. rafinesquii*. The larvae had a light neon-bluish colour when exposed to UV-light, after freezing.

### Morphology

**Figure 3:**
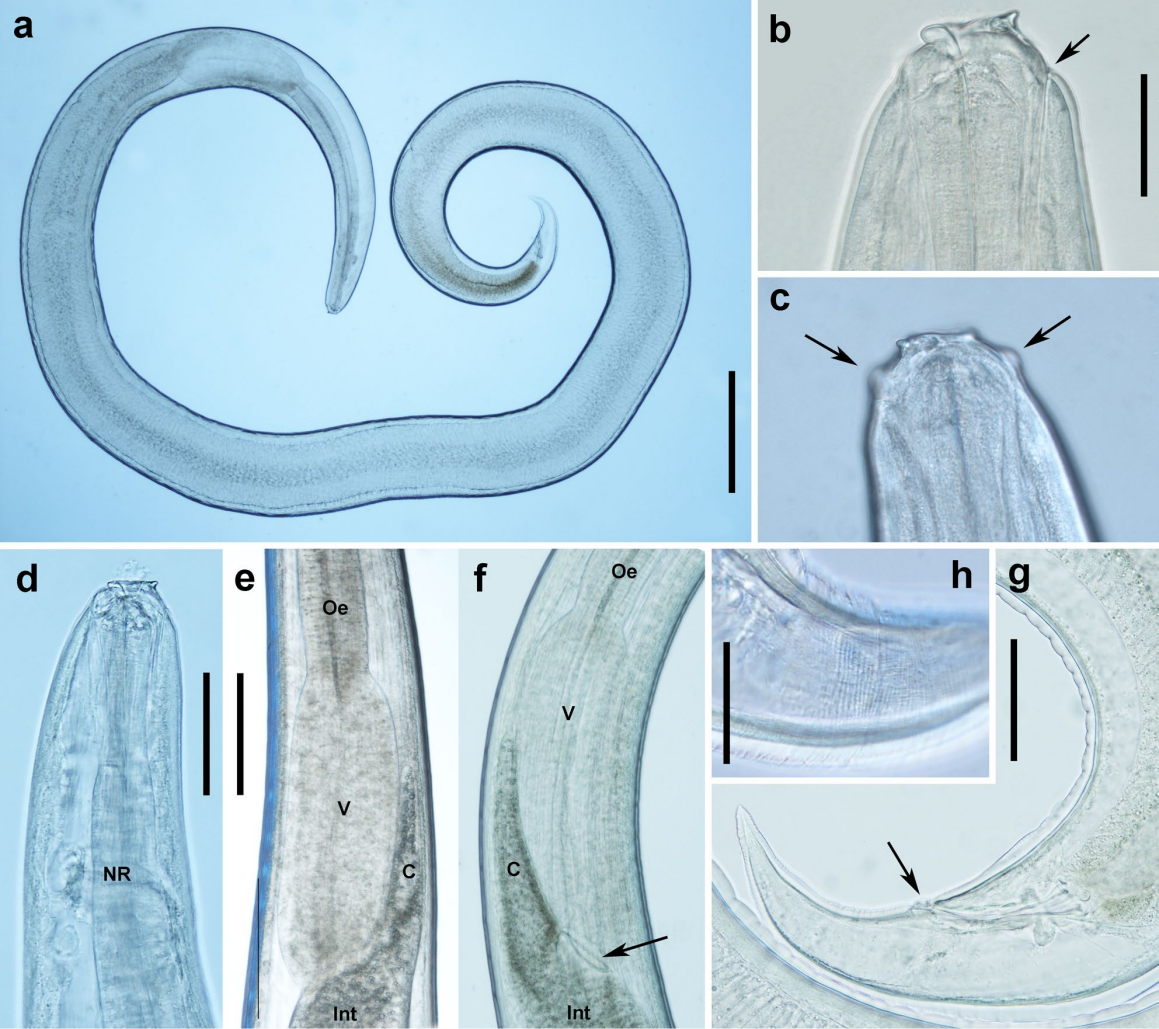
Digital photographs of *P. ceticola* L3 from Macaronesian deepwater fishes. (**a**) Entire *P. ceticola* larva from *Diaphus mollis*. (**b**) Anterior end showing the ventral boring tooth connected to the cuticular ridge, arrow indicates the excretory pore. (**c**) out-of-focus view of the anterior end, showing bulbs (arrows). (**d**) Anterior end showing nerve ring (NR). (**e–f**) ventricle region, showing ventricle (V) and caecum (C), intestine (Int), and oesophagus (Oe). Arrow in (**f**) indicates aperture intestine-ventricle. (**g**) Tail region, arrow indicating anus. (**h**) Part of tail, showing transverse cuticular striation. Specimens were collected from *D. mollis* (a,b,d,f,g) and *D. rafinesquii* (c,e,h). Scale bars: (**a**) 500 µm, (**b**, **c)** to same scale 50 µm, (**d**) 100 µm, (**e**, **f**) to same scale 200 µm, (**g**) 100 µm, (**h**) 50 µm.

Larvae were small and pale, with a thick-set appearance (Fig. 3, Table 4). The body was widest at the middle and posteriorly. Body length: max width ratio was 22—36:1 (mean 28 ± 4, *N* = 27). The cuticle was smooth, but with inconspicuous transverse striae which were most evident in tail. At anterior end, lip anlagen were visible through the cuticle, and associated with surface bulbs (Fig. 3c). Prominent conical boring tooth at the anterior extremity between ventro-lateral lip anlagen, projecting anteroventral at an angle of about 130° (115°–145°) to main axis. The boring tooth base gives rise to a circumoral cuticular ridge (Fig. 3b). The dorsoventral extent of this ridge from the boring tooth tip was 40.8 ± 3.2 µm (mean ± SD) (range = 36–49 µm; *N* = 25). The excretory pore was ventrally located, near the base of the boring tooth. The oesophagus was clavate, widest posterior. The nerve ring was positioned within the anterior 25 (20–27) % (mean (range); *N* = 12) of the oesophagus length. The oesophagus length constituted 10 (9–13) % (mean (range)) of the body length. The ventricle was prominent, wider than the posterior oesophagus; oesophagus to ventricle length ratio was 1.5–2.6:1 (2.0 ± 0.2; *N* = 27). The caecum was normally shorter than the ventricle, averaging 74% of its length (range = 50–119, SD = 18). The tail was elongate conical, curved ventrally, pointed and without a distinct mucron (Fig. 3–G).

**Table 4:** Measurements of *P. ceticola* L3 from seven Macaronesian meso- and bathypelagic fish species.

Measurement	N	Mean	SD	Min–Max
Total length (mm)	27	9.1	1.1	7.0–11.7
Maximum width	31	319	42	217–385
Oesophagus length	28	978	126	803–1472
Ventricle length	28	485	79	385–703
Ventricle width	26	154	25	109–199
Caecum length	27	360	98	197–637
Tail length	30	200	20	155–237

*N* = number of measurements, *SD* = Standard deviation. Measurements in µm unless specified.

### Molecular identification

The ITS sequences (801–842 bp) obtained from 13 *Terranova* type larvae were 100% identical. However, ambiguous positions (i. e. double signals) were seen in the sequences from five of these worms. The *cox*2 sequences (570–580 bp) obtained from 18 *Terranova* type larvae showed 97.1–99.4% identity. With a single exception (in DiRa38), all substitutions were silent. The *cox*2 sequences were 96.9% to 97.9% similar to a *Pseudoterranova ceticola cox*2 sequence from a Caribbean Sea *K. breviceps* (GenBank accession number DQ116435). Blast searches with the ITS sequence revealed 99.6–100% identity to sequences from adult worms (found in kogiid whales) or larvae (from marine fish and agnathans) identified as *Anisakis* sp. (see Supplementary file: Table S1, and further comments at discussion section). In addition, the ITS sequence of a *P. ceticola* larva from the Tanzanian fish *Saurida undosquamis* (ON128286)^19^ was 100% identical. Sequences of the presently identified *P. ceticola* L3 were deposited in GenBank with the accession numbers (ITS: OP352234- OP352246) and (*cox*2: OP380493-OP380510) (see also Supplementary file: Table S2).

### Phylogenetic analyses

Phylogenetic analyses were performed on ITS rDNA and mt DNA *cox*2 datasets.

**Figure 4:**
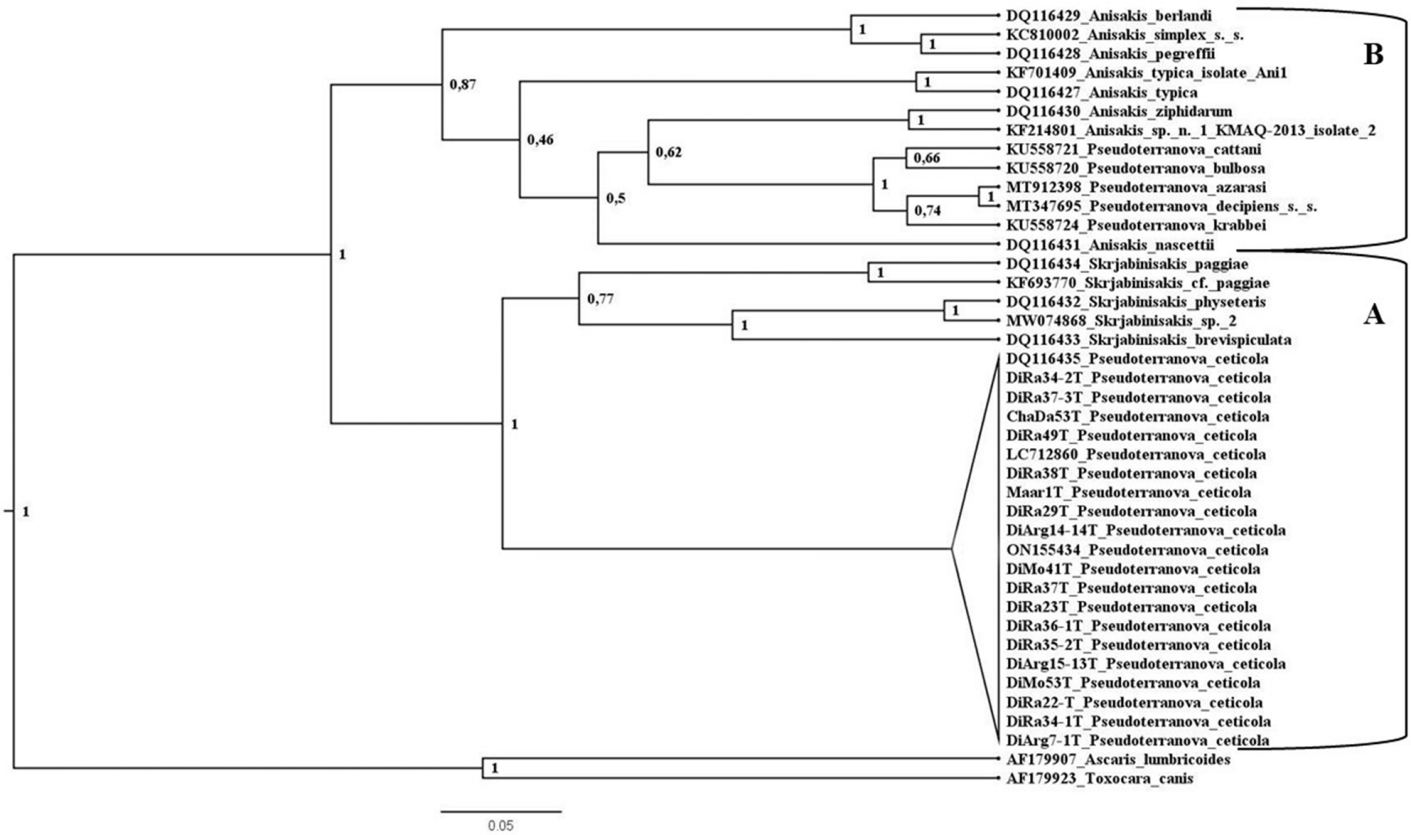
Phylogenetic tree from Bayesian inference based on *cox*2 sequences. **A**: clade **A**; **B**: clade **B**. Figure 4 was created in Figtree v1.4.4 (http://tree.bio.ed.ac.uk/software/figtree/).

In the *cox*2 BI tree, adult *P. ceticola* from *K. breviceps* (DQ116435), larva from the fish *S. undosquamis* (ON155434), larva from the fish *Katsuwonus pelamis* (LC712860) and the sequences of the present *Terranova*-like larvae group together in a well-supported clade, representing a sister group to a clade with *Skrjabinisakis physeteris*, *S. brevispiculata* and *S. paggiae* (until recently belonging to the genus *Anisakis* (see^20,21^) and related sequences (Fig. 4 and see also Figure S1 at Supplementary files which details the intraspecific variations and relationships of *P. ceticola*). The major clade (Clade A) with these two subclades is a well-supported sister group to a clade containing the *A. simplex* complex (*A. simplex* (*s.s.*)), *A. pegreffii*, *A. berlandi*), *Anisakis typica*, *Anisakis nascettii*, *Anisakis ziphidarum* and *Pseudoterranova* spp. from pinnipeds (*P. azarasi*, *P. bulbosa*, *P. cattani*, *P. decipiens* (*s .s.*) and *P. krabbei*) (Clade B).

**Figure 5:**
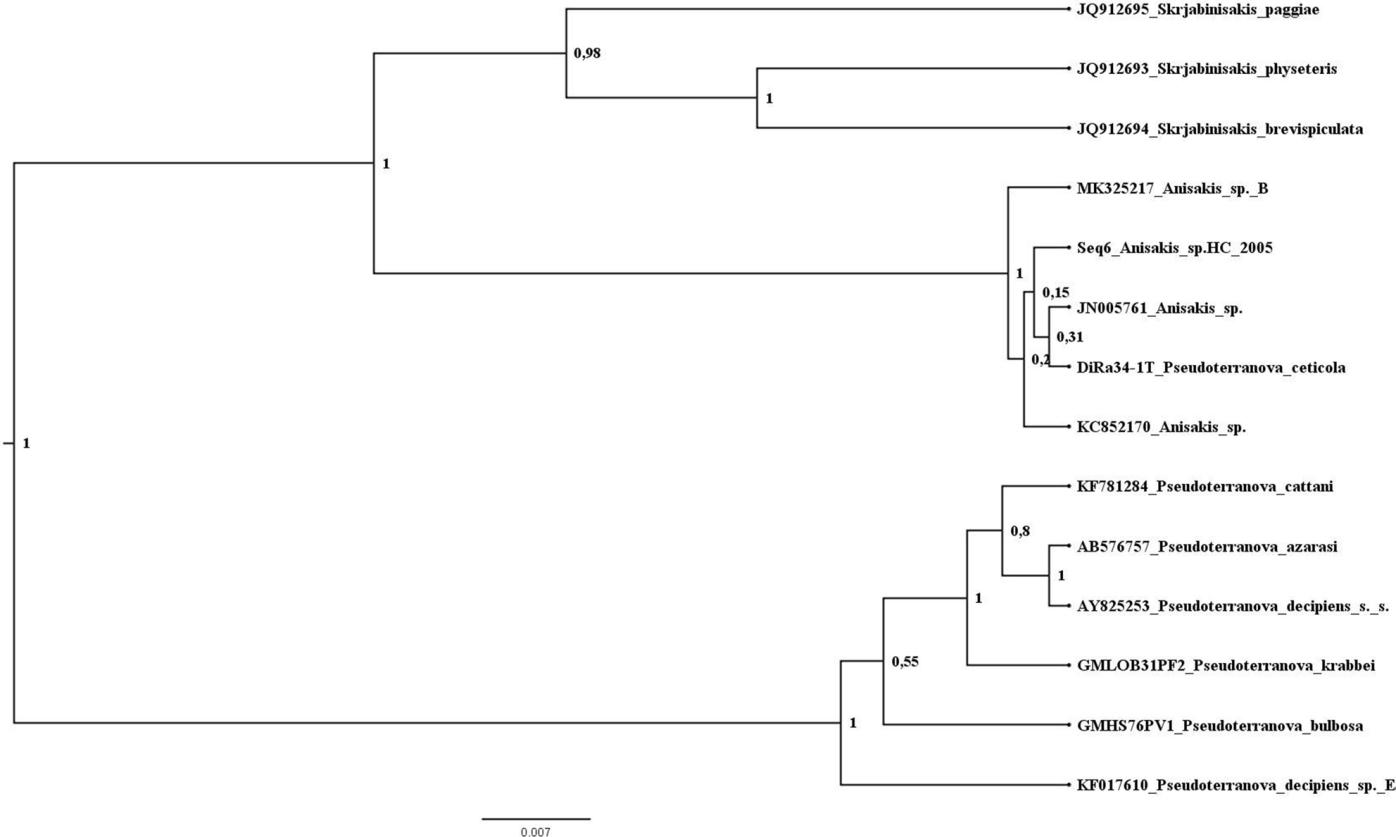
Unrooted tree from Bayesian inference based on ITS sequences. Figure 5 was created in Figtree v1.4.4 (http://tree.bio.ed.ac.uk/software/figtree/).

The unrooted tree obtained based on the ITS region sequences also supported Clade A and its two subclades (Fig. 5). Again, the pinniped *Pseudoterranova* spp. grouped separately. Also, the sequences of the present *Terranova*-like larvae grouped with larval and adult genotypes of worms identified as belonging to genus *Anisakis*, from fish and kogiid whales. These included worms from an Australian *K. breviceps* and a Philippine *K. sima*, a larva from the teleost fish *Pagellus bogaraveo* from Madeira and from the fish *Epinephelus areolatus* from Indonesia.

## Discussion

Genus *Terranova* was erected for a New Zealand shark parasite, *T. antarctica*^22,23^. Later additions of *Terranova* spp. represent further parasites from elasmobranchs, but also parasites from teleosts, crocodilians, colubrid snakes and marine mammals (reviewed by^4^). Attempts have been made to split the genus, and now the best-known species from poikilotherms (i. e. elasmobranchs, teleosts and reptiles) are allocated to genera *Euterranova*, *Neoterranova* or *Pulchrascaris*^4^. Several lesser known species are retained in *Terranova *sensu lato (species inquirenda, see^4^). Genus *Phocanema* was proposed for *Porrocaecum decipiens*^24^*,* a *Terranova*-like pinniped parasite that subsequently was shown to represent several cryptic species (see^3,25^)*.* A new genus, *Pseudoterranova*^26^*,* was proposed for *Terranova kogiae* from an Australian kogiid whale *Kogia breviceps*^27^, based on an erroneous interpretation of the excretory pore position^28^. Gibson^28^ corrected this error, but retained and redefined *Pseudoterranova* on the basis of the anterior extent of the glandular left filament of the excretory system. This genus now contains all the *Terranova*-like species from homeotherms, *P. kogiae* and *P. ceticola* from whales, and several species from pinnipeds, with *Phocanema* as a synonym.

The presently described nematode larvae were all morphologically similar, thus resembling a single morphotype. They show morphological characters shared by genera *Terranova *sensu lato, *Pseudoterranova* and *Pulchrascaris*, such as an excretory pore at the base of the ventral lip and the presence of an intestinal caecum. Recently, *Terranova* sp. type 1 and 2 larvae sensu Cannon^8^ from teleost paratenic hosts have been molecularly found to include *Pulchrascaris australis* and *Terranova pectinolabiata* (now *Euterranova pectinolabiata*) from Australian shark definitive hosts, respectively^4,8,29,30^. Gonzalez-Solis et al.^31^ used morphology alone to identify *Terranova* type 1 larvae from Hawaiian fishes as *Pulchrascaris* sp. In general, larvae of *Pulchrascaris* spp. and *Terranova* spp. that are present in teleost paratenic hosts and mature in elasmobranchs, show very long and slender ventricles, alongside an even longer caecum, and they have conical pointed tails without mucron^5,8,31,32^. The *Terranova*-like larvae described in the present study however have shorter more oval ventricles, accompanied by caeca that rarely exceed the ventricle in length. These characteristics are shared with larvae of *Pseudoterranova* spp. from seals and sea lions. However, the tails of the *Pseudoterranova* spp. larvae of pinniped infecting species differ from those of *P. ceticola* in being generally short and rounded with a mucron^10,33–35^. In addition, the boring tooth inclines ventrally and is prominent, different to those present in *Pseudoterranova* spp. from pinnipeds which are straighter and comparatively smaller^35–38^. The larvae presented in this study also appear to be considerably smaller in size, reaching only up to 12 mm in body length, compared to *P. decipiens s.l.* (10–60 mm), *P. cattani* (17–43 mm) or *P. decipiens sp. E* (20–38 mm)^6,33–36,38,39^. A most prominent character that distinguish the present larvae from pinniped *Pseudoterranova* spp. is the circumoral cuticular ridge connected with the boring tooth. This character also distinguishes them from most *Terranova *sensu lato larvae, that may represent elasmobranch parasites. However, Deardorff et al. found a *Terranova* larval type in Hawaiian teleosts, designated *Terranova* sp. type HA^40^. Those larvae fit the larvae presented in here in most aspects including caecum:ventricle length ratio, tail shape and the presence of an anterior circumoral ridge (Table 5). The *Terranova* sp. type HA larvae were found to survive in and penetrate the stomach wall of rats, demonstrating a zoonotic potential^40,41^. This suggests that they mature in a homeotherm, i. e., belong in genus *Pseudoterranova*. Similarly, Kuramochi et al. described *Pseudoterranova* cf. *ceticola* larvae recovered from stranded Japanese *K. breviceps* and the Risso’s dolphin (*Grampus griseus*) (see Table 5)^42^, and those larvae fit in most aspects the present larvae and the *Terranova* sp. type HA of Deardorff et al.^40^, thus suggesting to be conspecific. Most recently, a single *P. ceticola* larva from the scombrid fish *K. pelamis* in Japan was briefly described ^21^ (see Table 5). The larva, even though smaller, resembles in most aspects to our larva, however, authors did not refer to the circumoral cuticular ridge, which is apparent in their Fig. 1A ^21^, as the most prominent character of *P. ceticola* L3.

**Table 5:** Comparison of *P. ceticola* L3 with other similar larvae (range and mean in mm) and immature worms from kogiid whales.

L3 fishes				Immature worms, final host		
Designation	*P. ceticola* (this study)	*Terranova* type HA	*P. ceticola*	*P. ceticola*	*P.* cf. ceticola*	*Pseudoterranova* sp.
Host	See Table 1	*Aprion virescens*	*K. pelamis*	*K. breviceps*	*K. breviceps* and *Grampus griseus*	*K. breviceps*
Place	Macaronesia	Hawaii	Japan	Yucatan	Japan	Brazil
Body L	7.0–11.7 (9.1)	7.0–11.0	5.9	10.1–13.6	6.45–9.00	7.4–10.2 (8.2)
Oesophagus L	0.80–1.47 (0.98)	0.70–1.50		1.30–1.40	0.89–1.02	0.87–1.45 (1.12)
Ventricle L	0.39–0.70 (0.49)	0.42–0.57	0.38	0.40–0.50	0.40–0.58	0.43–0.53 (0.47)
Caecum L	0.20–0.64 (0.36)	0.30–0.52	0.49	0.50–0.60	0.36–0.53	0.50–0.53 (0.52)
Tail L	0.16–0.24 (0.20)	0.11–0.28	0.16	0.10–0.20	0.11–0.15	0.09–0.20 (0.14)
Reference	Present study	^43^	^21^	^44^	^42^	^45^

*Reported as third larval stage.

The present larvae are molecularly identified as *Pseudoterranova ceticola*, a parasite of kogiid whales. That finding suggests that the *Terranova* sp. type HA larvae are also likely *P. ceticola* larvae. *Kogia* spp. are common around the Hawaii islands (see Bloodworth and Odell^46^). *Pseudoterranova ceticola* was originally described from *Kogia sima* stranded in Mississippi, USA^47^. Another species, *P. kogiae* was described from *K. breviceps* in Australia. *Pseudoterranova kogiae* differs from *P. ceticola* by having more pairs of caudal papillae present in the males and by having three transverse rows of plectanes which are absent in *P. ceticola*^27,47,48^. These species appear to show a caecum equal or longer than the ventricle, even in juvenile specimens of *P. ceticola* reported from the final hosts (Table 5)^27,42,44,47^. Therefore, the relationship between caecum length and ventricle length in larvae from intermediate or transport hosts may not reflect the adult morphology, contrary to the arguments of González Solís et al.^31^. In addition, these species from kogiid whales are small compared to adult males maturing in pinnipeds. *Pseudoterranova ceticola* and *P. kogiae* ranged between 12 and 26 mm and 20–30 mm long, respectively, whereas the mean ± SD (range) for *P. decipiens s.s.*, *P. krabbei*, *P. bulbosa*, *P. cattani* and *P. azarasi* were reported as 44 ± 7 and 48 (42–54), 36 ± 3 and 35 (31–43), 47 ± 5 and 48 ± 7, 40 ± 9 (26–62) and 49 ± 3 mm long, respectively^27,42,44,47,49–52^.

The present identification relies on the high identity of the *cox*2 sequences with the reference sequence of *P. ceticola* collected from a Caribbean Sea *K. breviceps*^53^. Similarly, several *P. ceticola* collected from *K. breviceps* and *K. sima* stranded in Puerto Rico and Florida (USA) were morphologically and/or molecularly identified by sequencing analysis of the mtDNA *cox*2^54,55^, so the association of this genotype with *P. ceticola* should not be in doubt. However, the herein presented ITS1-5.8S-ITS2 sequences from the same *P. ceticola* larvae specimens showed 100% identity to sequences from some worms identified as *Anisakis* sp.^56–60^ (see Table S1 at supplementary file). It is probable that this occurred due to the lack/impossibility of a morphological examination (probably hampered by a poor condition of worms in those studies) revealing the characteristic caecum of *P. ceticola*, in combination with a GenBank match for *Anisakis* sp. In relation to this, in cleared specimens the caecum may become very pale and not easy to distinguish, hence it might be overlooked (per. obs., see also^61^). The diagnostic RFLP pattern of *P. ceticola* with the *Hha*I restriction enzyme produce 4 bands, of about 80, 180, 200 and 400 bp length, and a single undigested band of about 1000 bp with *HinfI*^55,62^. However, this pattern has also been obtained with worms that may have been erroneously identified as *Anisakis* sp.^57–59,63,64^, leading to confusion. A list of the anisakids likely representing *P. ceticola* but identified as *Anisakis* sp. is provided in the Supplementary file Table S3.

Clearly, one reason for this confusion is the lack of a morphological account of *P. ceticola* larvae, which we provide here. However, trustable sequence information from *P. kogiae* is not available. Either *P. kogiae* and *P. ceticola* are two valid species, or they are synonymous. Gibson examined the types of *P. kogiae* and also had at hand specimens of *P. ceticola* from South African *K. breviceps*, and seems to consider them valid species^28^. Shamsi et al.^65^ provided an ITS region sequence (MK325217) of an “*Anisakis* sp.” from an Australian *K. breviceps*, interestingly being the same type host and geographical area where *P. kogiae* was described^27^. The sequence was considerably different to our *P*. *ceticola* sequences by having a unique 3 bp insert plus 2 substitutions (see also Fig. 5). Clearly, more research is necessary in order to clarify the relationship between these *Pseudoterranova* spp.

Phylogenetic analysis obtained from the mtDNA *cox*2 sequences, suggests that the clade with *P. ceticola* sequences is closely related to a clade formed by *S. paggiae*, *S.* cf. *paggiae*, *S. brevispiculata*, *S. physeteris* and *Skrjabinisakis* sp. 2 (species which until most recently belonged to the genus *Anisakis*^20,21^). The BI analysis of the ITS region also supports that *P. ceticola* is closely related to the former *S. physeteris* complex clade. These results are similar to those by Cavallero et al.^28^ and most recently to those by Takano & Sata^21^. The monophyly of the clade formed by *P. ceticola* and *S. paggiae*, *S. brevispiculata* and *S. physeteris* was suggested by Cavallero et al. based on phylogenetic analyses of the *cox*2 and ITS regions, as found here. However, Takano & Sata found the species *Neoterranova caballeroi* from reptiles as the most related species to *P. ceticola* which raises concerns^21^. We have performed an additional BI analysis including the *cox*2 sequence of *N. caballeroi* (AF179921), and *N. caballeroi* was placed as an offshoot of Anisakinae species (i. e. *Anisakis*, *Skrjabinisakis* and *Pseudoterranova*), which we believe is more congruent with the ecology and morphology of this parasite (see figure S2 at Supplementary files).

In this study, *P*. *ceticola* was collected from fishes which distributions span the meso- and bathypelagic zones, caught off Cape Verde, Canary Islands and Madeira. *Pseudoterranova ceticola* larva in the deep-water shark *Centrophorus squamosus* taken off Madeira was also reported by Costa et al.^62^, who identified it by ITS-RFLP but provided no sequence information. Recently, Cipriani et al. identified a single *P. ceticola* larva from the reef-associated fish *S. undosquamis* caught between 100–600 m depth off the coast of Tanzania, by sequencing analysis of the ITS rDNA and mtDNA *cox*2 sequences^19^. Most recently, a single *P. ceticola* larva from the scombrid fish *K. pelamis* was also identified in Japan^21^. It appears possible that the former fish species, i. e. *C. squamosus*, *S. undosquamis* or *K. pelamis*, could have acquired *P. ceticola* though predation upon parasitized meso/bathypelagic fish.

It appears then that *P. ceticola* may have different host specificity depending on life stages, being a host specialist in the final host (i. e. kogiid whales), and generalist in the second intermediate or paratenic host (i. e. fishes). Adult *P. ceticola* has only been found in kogiid whales (i.e. *K. sima* and *K. breviceps*) suggesting stenoxeny at the final host level. *Pseudoterranova ceticola* was reported in *K. sima* from the Gulf of Mexico, Japan, Caribbean and SE Atlantic coasts of USA, and from *K. breviceps* in the same geographical region (presumably only as larvae in Japan), Atlantic Canada, NW Spain and South Africa^28,42,44,47,48,53–55,66–68^. In addition, *Pseudoterranova* sp. (as *Terranova* sp.) has been reported in *K. breviceps* from Brazil, Pacific Gulf of California (Mexico) and France, and in *K. breviceps* and *K. sima* from the Caribbean region^45,68–70^. *Pseudoterranova* cf. *ceticola* L3 has been reported from two Japanese *G. griseus* and *Pseudoterranova* sp. has been reported from Caribbean pygmy killer whale (*Feresa attenuata*)^42,68^.

Thus, *P. ceticola* has so far been found in temperate waters of western and eastern Atlantic and Pacific Oceans (see above and Table S3), a distribution apparently overlapping that of its final kogiid hosts^71^.In addition, kogiids have been reported stranded or observed in Macaronesia areas^46,71–73^. Contrarily, *Pseudoterranova* spp. from pinnipeds are mainly distributed in Boreal and Austral waters where their final hosts thrive^74^.

Our results suggest that the life cycle of *P. ceticola* occur in the mesopelagic and bathypelagic realm. In addition, the parasite also appears to occur in benthopelagic, demersal and even reef-associated fish hosts (see Table S3). Contrarily, primarily neritic, benthic and demersal fishes appears to be involved in transmitting *Pseudoterranova* spp. to pinnipeds^3,6,36,74–77^. Indeed, it has been observed that *P. decipiens s.l.* larvae hatched from eggs adhered to the substrate by their tails^76,78^, being eaten by benthic meiofauna (mainly copepods) first intermediate hosts leading to transmission up a benthic food-web^76,79^.

There are some studies which have indicated that meso- and bathypelagic fish are prey for *K. breviceps* and *K. sima*^46,72,80^. West et al.^80^ analysed the stomach content of stranded *K. breviceps* of the Hawaiian archipelago and identified among others *D. argenteus*, *Diaphus* sp. and *E. pelecanoides*, which are species that were found infected by *P. ceticola* in the present study. The parasite might also be transmitted through the food web from mesopelagic fish to squids (and other fishes) and then to the whales, since mid and deep-water cephalopods are also known as a very important part of the diet of these kogiids^71,72,80^.

## Conclusions

*Pseudoterranova ceticola* third-stage larva (L3) was herein fully described for the first time. The parasite was recovered from meso- and bathypelagic fishes from off Macaronesia archipelagos (NW Africa). L3 were small, pale, with a thick-set appearance and bluish when exposed to UV-light after thawing. Ventricle morphology, presence of a caecum, tail shape and the presence of a circumoral cuticular ridge extending dorsally from boring tooth are morphological characteristics that aid identification. *Pseudoterranova ceticola*, which has kogiid (Physeteroidea) whales as final hosts, is related to *Skrjabinisakis* spp. (whose species formerly belonged to the genus *Anisakis*) maturing in physeteroid whales, rather than to *Pseudoterranova* spp. from pinnipeds. This is evidence that genus *Pseudoterranova* may have to be split.

## Supplementary Information

Supplementary Information.

## Data Availability

Data supporting the conclusions of this article are included within the article and its supplementary files. The DNA sequences of the *P. ceticola* specimens identified were deposited in the public sequence repository GenBank (NCBI National Center for Biotechnology Information—https://www.ncbi.nlm.nih.gov/genbank), and their accession numbers can be found in the present manuscript (Table 2 and 3) and/or supplementary files (Table S2).
