# Supplementary material for: Characterization of Pseudoterranova ceticola (Nematoda: Anisakidae) larvae from meso/bathypelagic fishes off Macaronesia (NW Africa waters)
Source: Sci Rep. 2022 Oct 21;12:17695. doi: 10.1038/s41598-022-22542-0 (PMC9587057; doi:10.1038/s41598-022-22542-0)
Supplement: Supplementary file 1 — Supplementary Information. [file 41598_2022_22542_MOESM1_ESM.docx]

# Supplementary information

**Table S1**. Blast scores of the present ITS sequences of *P. ceticola* matching exactly or closely to unidentified *Anisakis* sp. in GenBank.

| **Acc. num.** | **Parasite** | **Host** | **Location** | **Perc. Ident.** | **Ref** |
| --- | --- | --- | --- | --- | --- |
| KC342894 | *Anisakis sp.* | *K. sima* | Philippines | 100% | Quiazon et al [53] |
| KC342892 | *Anisakis* sp. | *K. sima* | Philippines | 100% | Quiazon et al [53] |
| EU718474 | *Anisakis sp.* | *Hoplostethus cadenati* | Mauritania | 100% | Kijewska et al [52] |
| KC342893 | *Anisakis sp.* | *K. sima* | Philippines | 100% | Quiazon et al [53] |
| JX523712 | *Anisakis sp.* | *Carcharhinus* sp. | South China Sea | 100% | Zhang et al [54] |
| KT964236 | *Anisakis* sp. | *Eptatretus* spp. | Taiwan | 100% | Luo et al. [55] |
| KC852166 | *Anisakis sp.* | *K. sima* | Philippines | 100% | Quiazon et al [53] |
| KC852165 | *Anisakis sp.* | *K. sima* | Philippines | 100% | Quiazon et al [53] |
| KC852168 | *Anisakis* sp. | *K. sima* | Philippines | 100% | Quiazon et al [53] |
| KC852171 | *Anisakis* sp. | *K. sima* | Philippines | 100% | Quiazon et al [53] |
| KC852167. | *Anisakis* sp. | *K. sima* | Philippines | 100% | Quiazon et al [53] |
| KC852164 | *Anisakis* sp. | *K. sima* | Philippines | 100% | Quiazon et al [53] |
| KC852169 | *Anisakis* sp. | *K. sima* | Philippines | 100% | Quiazon et al [53] |
| MF668740 | *Anisakis* sp. | *Coryphaena hippurus* | South Carolina, USA | 100% | Quiazon et al (unpublished) |
| MF768443 | *Anisakis* sp. | *Katsuwonus pelamis* | South Carolina, USA | 100% | Quiazon et al (unpublished) |
| MF668739 | *Anisakis* sp. | *Thunnus atlanticus* | South Carolina, USA | 100% | Quiazon et al (unpublished) |
| KC121370 | *Anisakis* sp. | *Lepturacanthus savala* | Indonesia | 100% | Kuhn et al [56] |
| JN005761 | *Anisakis* sp. | *Pagellus bogaraveo* | Madeira | 99.88% | Hermida et al [59] |
| KC852170 | *Anisakis* sp. | *K. sima* | Philippines | 99.75% | Quiazon et al [53] |
| MF668741 | *Anisakis* sp. | *Coryphaena hippurus* | South Carolina, USA | 99.74% | Quiazon et al (unpublished) |
| KY524195 | *Anisakis* sp. | *Sufflamen fraenatum* | Indonesia | 99.63% | Palm et al [88] |
| MK325217 | *Anisakis* sp. B | *K. breviceps* | Australia | 99.34% | Shamsi et al [61] |

**Table S2:** GenBank accession numbers (i. e. ITS number and *cox*2 number) of *Pseudoterranova ceticola* L3 isolates from meso/bathypelagic fish species of Macaronesian waters (NW Africa).

| **Host** | **Isolate** | **ITS number** | ***cox*2 number** |
| --- | --- | --- | --- |
| *Chauliodus danae* | ChaDa53T | OP352244 | OP380506 |
| *Eurypharynx pelecanoides* | EuPele13T | OP352245 | - |
| *Diaphus mollis* | DiMo53T | OP352234 | OP380494 |
|  | DiMo41T | OP352235 | OP380495 |
| *Diaphus rafinesquii* | DiRa23T | OP352236 | OP380496 |
|  | DiRa37T | OP352238 | OP380498 |
|  | DiRa34-1T | OP352239 | OP380499 |
|  | DiRa29T | OP352240 | OP380500 |
|  | DiRa35-2T | OP352241 | OP380501 |
|  | DiRa49T | OP352242 | OP380502 |
|  | DiRa34-2T | - | OP380503 |
|  | DiRa36-1T | - | OP380504 |
|  | DiRa38T | OP352243 | OP380505 |
|  | DiRa22T | - | OP380509 |
|  | DiRa37-3T | - | OP380510 |
| *Diretmus argenteus* | DiArg15-13T | OP352237 | OP380497 |
|  | DiArg7-1T | - | OP380508 |
|  | DiArg14-14T | OP352246 | OP380507 |
| *Maulisia argipalla* | Maar1T | - | OP380493 |

**Table S3.** Records of unidentified *Anisakis* sp. that based on the presently obtained molecular evidence likely represent *P. ceticola*. Abbreviations: Acc. num.= GenBank accession number; ID= identification; Ref.= References; ITS seq. = ITS sequence.

| **Acc. num.** | **Host** | **Place** | **Host habitat** | **Basis of corrected ID** | **Ref.** |
| --- | --- | --- | --- | --- | --- |
| EU718474 | *Hoplostethus cadenati* | Mauretania | Bathypelagic | ITS seq., PCR-RFLP | Kijewska et al. [52] |
| None | *Helicolenus dactylopterus* | Azores, Madeira, mainland Portugal | Bathydemersal | PCR-RFLP | Sequeira et al. [60] |
| JN005761 | *Pagellus bogaraveo* | Madeira | Benthopelagic | ITS seq., PCR-RFLP | Hermida et al. [59] |
| JX523712 | *Carcharhinus* sp., *Euthynnus affinis*, *Saurida elongata* | South China sea | _, Pelagic-neritic, demersal | ITS seq., PCR-RFLP | Zhang et al. [54] |
| KC121370 | *Lepturacanthus savala* | Indonesia | Benthopelagic | ITS seq. | Kuhn et al. [56] |
| KC342892-4, KC852164-71 | *Kogia sima* | Philippines | Continental shelf and slope in the epi and mesopelagic zones (see McAlpine [67]). | ITS seq. PCR-RFLP | Quiazon et al. [53] |
| None | *Epinephelus areolatus* | Indonesia | Reef-associated | ITS seq. | Kleinertz et al. [77] |
| KT964236 | *Eptatretus burgeri*, *E. yangi*, *E. sheni* | Taiwan | Demersal, demersal, bathydemersal | ITS seq. PCR-RFLP | Luo et al. [55] |
| KY524195 | *Sufflamen fraenatum* | Indonesia | Reef-associated | ITS seq. | Palm et al. [88] |
| MF668739-41, MF768443 | *Coryphaena hippurus*, *Katsuwonus pelamis, Thunnus atlanticus* | South Carolina | Pelagic-neritic, pelagic-oceanic, pelagic-oceanic | ITS seq. | Quiazon et al. (unpublished) |

**Figure S1.** Phylogenetic tree from Bayesian inference based on *cox*2 sequences, detailing the intraspecific variations and relationships of *P. ceticola*.


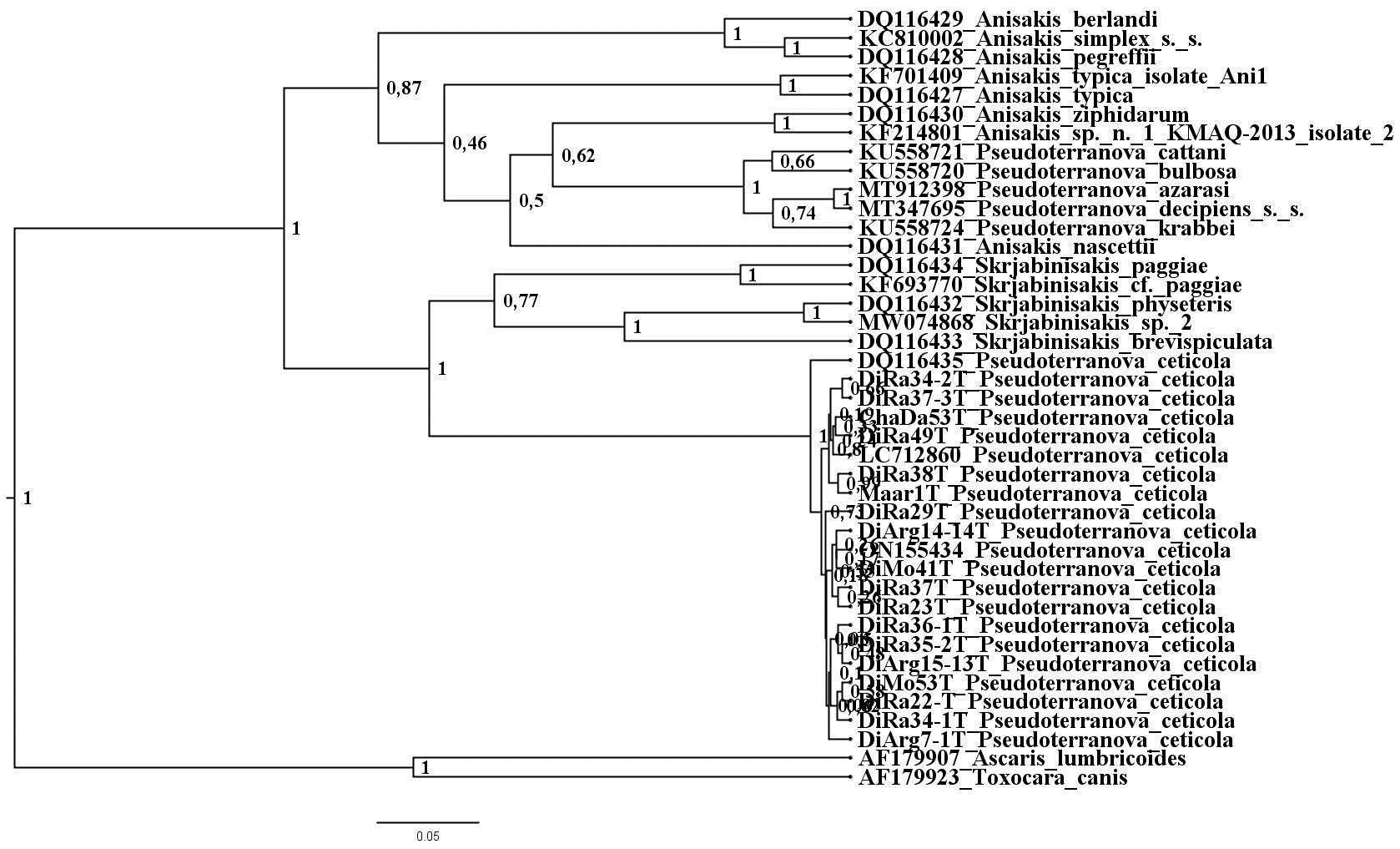


**Legend.** Figure S1 was created in Figtree v1.4.4 (http://tree.bio.ed.ac.uk/software/figtree/).

**Figure S2**. Phylogenetic tree from Bayesian inference based on *cox*2 sequences, including the sequence of *Neoterranova caballeroi* (AF179921).


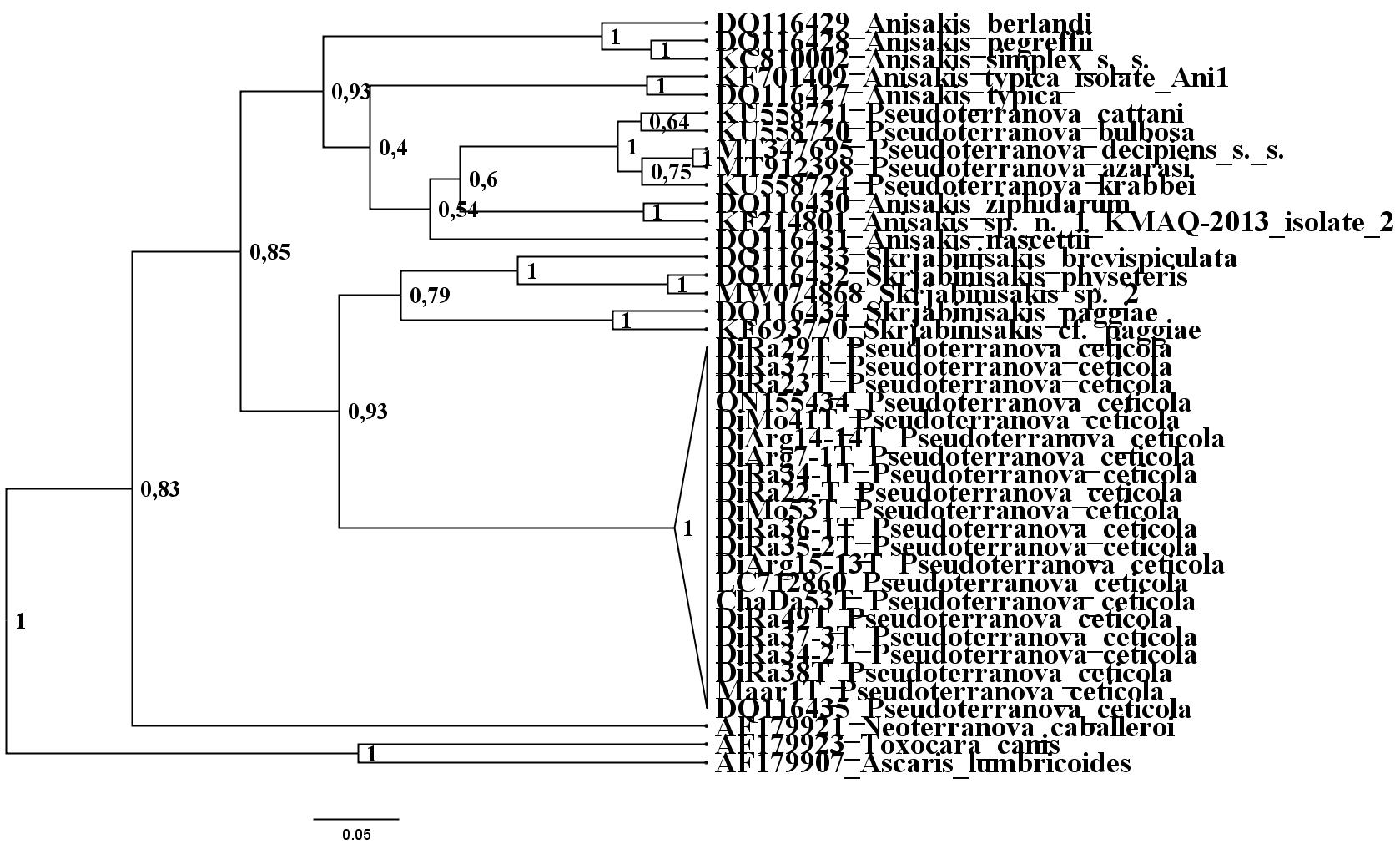


**Legend.** Figure S2 was created in Figtree v1.4.4 (http://tree.bio.ed.ac.uk/software/figtree/).
